# Microvesicles from the plasma of elderly subjects and from senescent endothelial cells promote vascular calcification

**DOI:** 10.18632/aging.101191

**Published:** 2017-03-08

**Authors:** Matilde Alique, María Piedad Ruíz-Torres, Guillermo Bodega, María Victoria Noci, Nuria Troyano, Lourdes Bohórquez, Carlos Luna, Rafael Luque, Andrés Carmona, Julia Carracedo, Rafael Ramírez

**Affiliations:** ^1^ Departamento de Biología de Sistemas, Facultad de Medicina y Ciencias de la Salud, Universidad de Alcalá, Alcalá de Henares, Madrid, Spain; ^2^ Departamento de Biomedicina y Biotecnología, Facultad de Biología, Química y Ciencias Ambientales, Universidad de Alcalá. Alcalá de Henares, Madrid, Spain; ^3^ Unidad de Anestesia, Hospital Universitario Reina Sofía/Universidad de Córdoba, Córdoba, Andalucía, Spain; ^4^ Instituto Maimónides de Investigación Biomédica de Córdoba (IMIBIC)/Hospital Universitario Reina Sofía/Universidad de Córdoba, Córdoba, Andalucía, Spain; ^5^ Departamento de Química Orgánica, Universidad de Córdoba, Edificio Marie Curie (C-3), Carretera Nacional IV-A, Km 396, E14014, Córdoba, Andalucía, Spain; ^6^ Departamento de Fisiología Animal (II), Facultad de Biología, Universidad Complutense de Madrid, Madrid, Spain; ^7^ Institute of Investigation, Hospital 12 de Octubre, Madrid, Spain

**Keywords:** aging, senescence, microvesicles, vascular calcification, endothelial cells, vascular smooth muscle cells

## Abstract

Vascular calcification is commonly seen in elderly people, though it can also appear in middle-aged subjects affected by premature vascular aging. The aim of this work is to test the involvement of microvesicles (MVs) produced by senescent endothelial cells (EC) and from plasma of elderly people in vascular calcification. The present work shows that MVs produced by senescent cultured ECs, plus those found in the plasma of elderly subjects, promote calcification in vascular smooth muscle cells. Only MVs from senescent ECs, and from elderly subjects' plasma, induced calcification. This ability correlated with these types of MVs' carriage of: a) increased quantities of annexins (which might act as nucleation sites for calcification), b) increased quantities of bone-morphogenic protein, and c) larger Ca contents. The MVs of senescent, cultured ECs, and those present in the plasma of elderly subjects, promote vascular calcification. The present results provide mechanistic insights into the observed increase in vascular calcification-related diseases in the elderly, and in younger patients with premature vascular aging, paving the way towards novel therapeutic strategies.

## INTRODUCTION

Vascular calcification is an undervalued risk factor for the appearance of cardiovascular disease (CVD). Regarded as a surrogate marker for atherosclerosis, a condition that frequently precedes coronary events [1], calcification is commonly seen in the vasculature of elderly subjects, and in middle-aged subjects with premature vascular disease associated to chronic kidney disease [2]. Hitherto, vascular calcification was thought to be a consequence of simple, physical mineral deposition in the vessel walls. New evidence, however, has revealed a highly regulated cellular response to be involved, and that calcification is the result of an imbalance between the inhibitors and inducers of Ca deposition [3]. The plasma calcium/phosphate (Ca/P) balance and the increase of proteins involved in bone formation (osteopontin, osteocalcin, certain proteoglycans, and bone morphogenetic protein 2 [BMP2]) [4]*,* among other factors, have all been found involved [3].

Microvesicles (MVs) –also named microparticles- are a subset of extracellular vesicles [5]. Recent studies have shown that MVs produced by a smooth muscle cells, can carry Ca as well as molecules (P and proteins) that act as calcification nucleation sites. They may therefore also be involved in initiating vascular calcification [3, 6]. Indeed, some MVs carry Ca-binding annexins, among them A2 and A6 [6, 7]. Endothelial senescence is known to be involved in the initiation of certain CVDs such as atherosclerosis and hypertension; the MVs produced by senescent ECs might therefore play an important role in their onset [8, 9]. Previous studies by our group have shown that microparticles produced by ECs in response to inflammatory stimuli, promote a calcifying response in vascular smooth muscle cells [10].

The aim of the present study was to determine: 1) whether MVs produced by senescent, cultured ECs, plus those found in the plasma of elderly subjects, promote calcification in vascular smooth muscle cells, and 2) to determine which contents of such MVs might be involved.

## RESULTS

### Quantification and characterization of plasma-derived microvesicles

**Figure 1:**
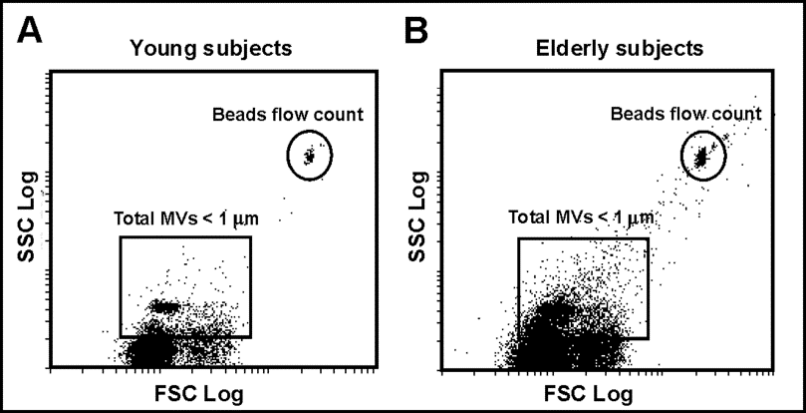
Plasma MVs assessed by flow cytometry (**A**) Representative dot plot showing log forward scatter (FSC) *vs*. log side scatter (SSC) localization of MVs in a young subject. The upper right gate shows the bead flow count, used as an index to count MVs in absolute terms. The lower left gate shows MVs smaller than 1 μm. (**B**) Representative dot plot showing localization of MVs in an elderly subject.

Flow cytometric analysis of the plasma-derived MVs from both the young and elderly subjects localized the MVs population around the forward scatter signal corresponding to particles with a diameter of around 1 μm (Fig. 1). Figure 1B shows that the plasma of the elderly subjects contained a greater number of total MVs.

As showed in Table 1, elderly subjects' MVs contained an increase total MVs (annexin A5+) compared with young subjects (6959±1187/μL vs 4868±547/μL; p<0.001). Additionally, significantly more of the elderly subjects' MVs carried annexin A5+/CD31+/CD42+ platelet-associated antigens (5254±1051/μL compared with 4176±661/μL of the young subjects' MVs; p=0.004). In addition, more carried endothelial-associated annexin A5+/CD31+/CD42- antigens (1850±338/μL compared with 692±260/μL; p<0.001).

**Table 1:** Demographic data and MVs (number /μL) from elderly and young subjects

Donors	Age (years) *Mean ± SEM (ratio)*	Gender, female % *(male/female)*	Total MV Annexin A5+	Total MV Annexin A5+	
				CD31+ CD42+	CD31+ CD42-
**Young** (n=15)	23.6 ± 2.6 (20-28)	53 % (7/8)	4868 ± 547	4176 ± 661	692 ± 260
**Elderly** (n=11)	78.9 ± 3.2*** (75-83)	55 % (5/6)	6959 ±1187***	5254 ± 1051**	1850 ± 338***

*** p<0.001 *vs* Young

** p<0.01 *vs* Young

### Quantification and characterization of HUVEC-derived microvesicles

**Figure 2:**
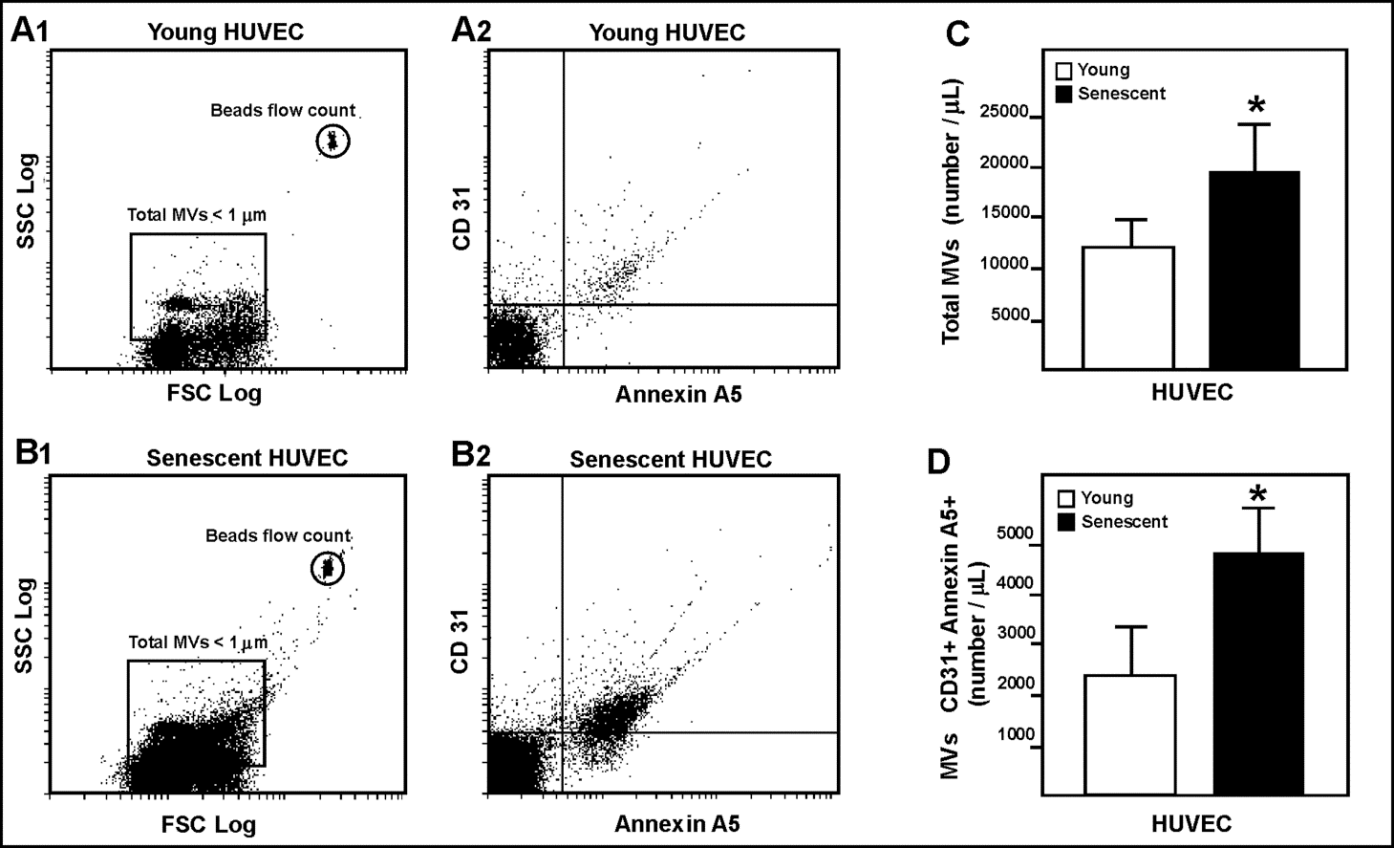
HUVEC-derived MVs assessed by flow cytometry Representative dot plots showing log forward scatter (FSC) *vs*. log side scatter (SSC) from young (**A1**) and senescent (**B1**) HUVEC. Representative dot plots showing annexin A5 and CD31 phenotype from young (**A2**) and senescent (**B2**) HUVEC. (**C**) Absolute number of MVs per μL of sample from young and senescent HUVEC; *p<0.05. (**D**) Absolute number of CD31+/annexin A5+ MVs per μL of sample; *p<0.05. Results are presented as the mean ± SD of 6 independent experiments.

Cytometric characterization revealed most of these MVs showing a similar distribution from both young and senescent endothelial cells (representing a diameter of 1 μm; Fig. 2A1 and 2B1). The number of total MVs in the culture medium of the senescent human umbilical vein endothelial cells (HUVEC) was greater than in that of the young cells (Fig. 2A2, 2B2 and 2C). These senescent HUVEC-derived MVs expressed more annexin A5+/CD31+ than those derived from young HUVEC (Fig. 2D). Based on the absence of fluorescent nuclear staining by acridine orange, only a small percentage of MVs (<10%) showed characteristics of apoptotic bodies.

### Microvesicles from senescent HUVEC and elderly subjects' plasma induce calcification in HASMC

**Figure 3:**
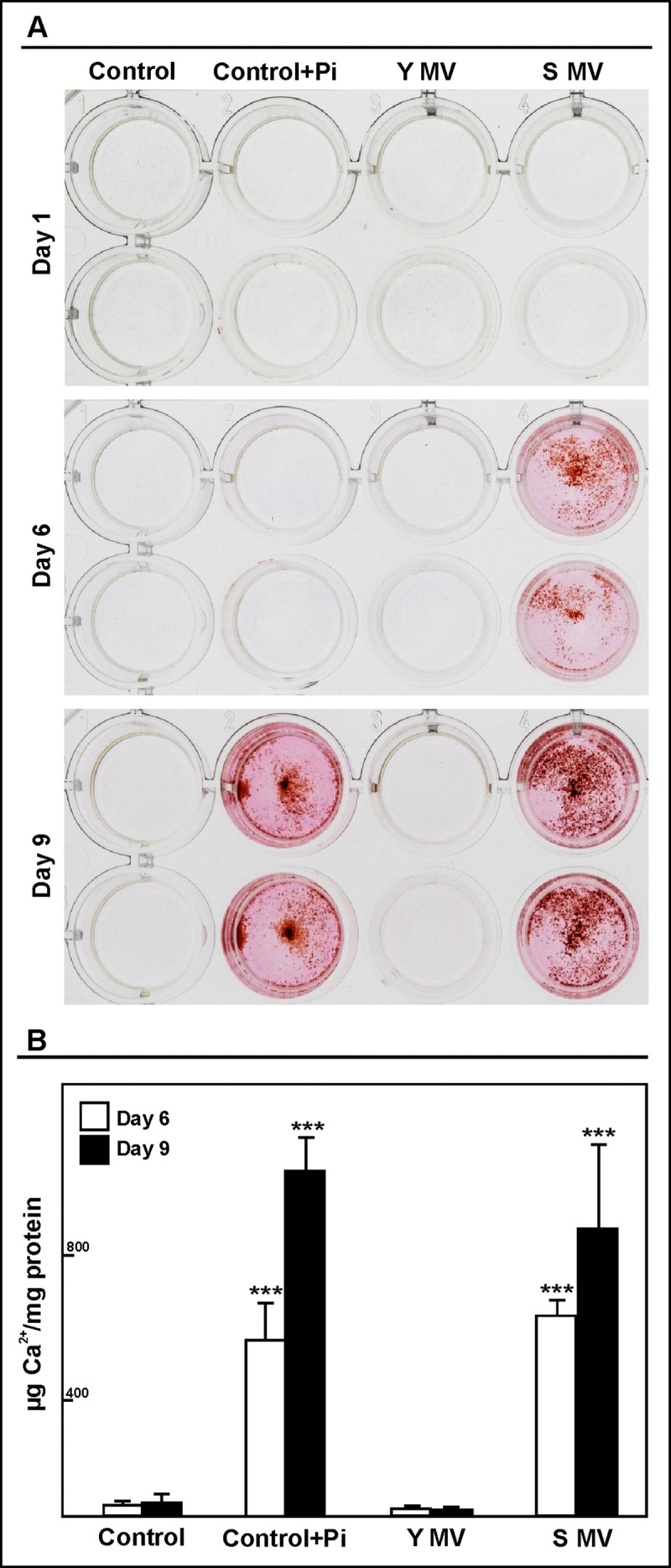
MVs from senescent HUVEC cells induced calcification in HASMC Cells were cultured with 50,000 MVs/mL from young (Y MV) or senescent (S MV) HUVEC for different times. (**A**) Qualitative calcification was stained with alizarin red. A representative experiment from six different experiments per duplicate is show. (**B**) Calcium content was determined by spectrophotometer by phenolsulphonephthalein dye at 6 and 9 days of treatment. The graphs present calcium content of the cells expressed as μg/mg protein. The data represent means ± SD of 3 independent experiments. *** p<0.001 vs control and Y MV at the same time.

To determine the ability of MVs to promote calcification, human aortic smooth muscle cells (HASMC) were cultured in the presence of MVs (50,000 MVs/mL) from senescent or young endothelial HUVEC, (Fig. 3A). Only the MVs from the senescent HUVEC induced calcification in the HASMC. In fact, alizarin red deposits were observed after 6 days of treatment. These deposits were not observed in HASMC grown with MVs from the young HUVEC. HASMC were incubated with inorganic P (Pi), a pro-calcific condition, as positive control; in the negative control HASMC were grown without MVs and Pi. This last control did not show red alizarin stain. A similar effect was observed using MVs (100,000 MVs/mL) from elderly and from young subjects.

In order to quantify the process of calcification, calcium content was analysed using a colorimetric method containing phenolsulphonephthalein dye (Figure 3B). The calcium content was significantly increased in HASMC after 6 and 9 days of treatment with senescent HUVEC MVs. A similar effect was observed in pro-calcific condition.

### Capture of HUVEC-derived microvesicles by HASMC

**Figure 4:**
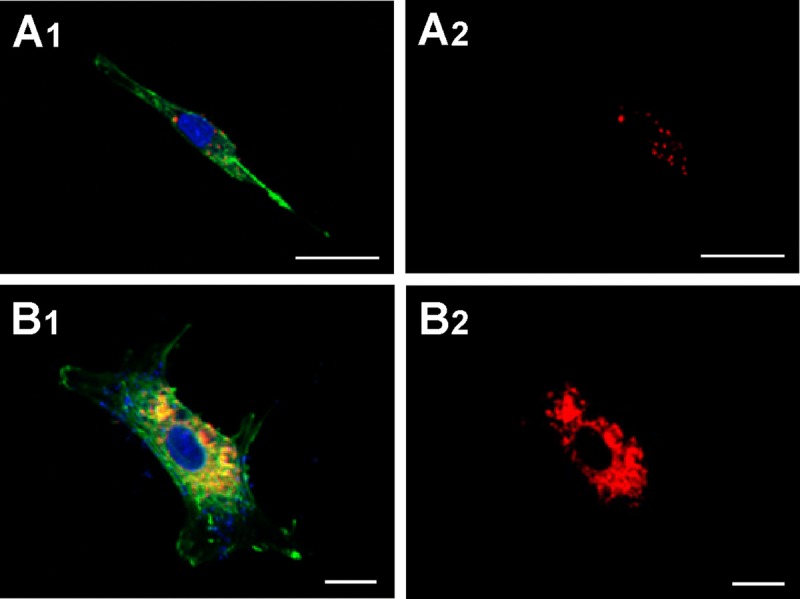
Capture of MVs by HASMC Cells were incubated for 48 h with MVs from young (panels **A1** and **A2**) or senescent HUVEC (panels **B1** and **B2**) stained with CellTracker CM-Dil PI (red) and then were stained with phalloidin (PKH67, green). Nuclei were stained with DAPI (blue). Fluorescence was evaluated by confocal microscopy. (A) Scale bar 25μm, and (B) scale bar 10 μm. A representative experiment from three different experiments is shown.

HUVEC-derived MVs were labelled with CellTracker CM-Dil PI (red) and cultured for 48 h with HASMC to determinate whether they promote calcification by adherence to the cell surface and/or by being endocytosed by the latter cells. After that HASMC were stained with PKH67 phalloidin (green). Confocal microscopy showed the majority of the MVs to locate inside the cytoplasmic compartment of the HASMC (Fig. 4). This was true for the MVs from both the young and senescent HUVEC.

### Senescent HUVEC-derived microvesicles carry Ca

**Figure 5:**
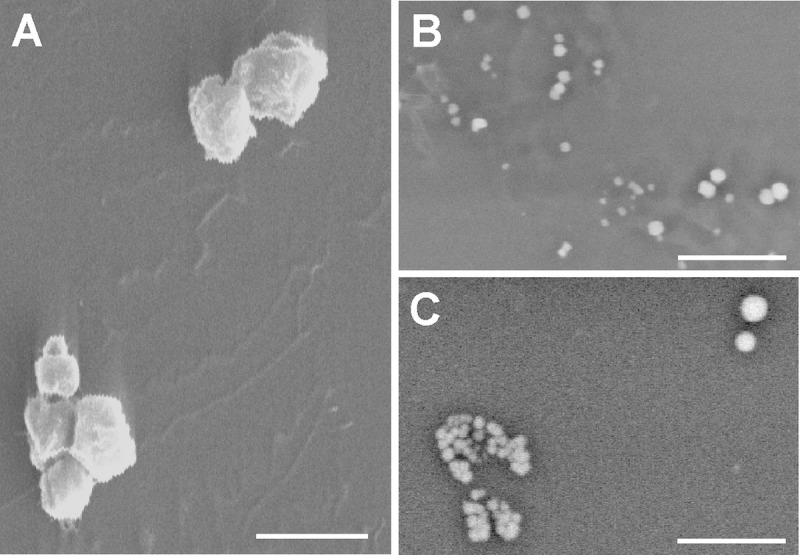
Scanning electron microscopy (**A**) Gold-palladium coated senescent MVs. Scale bar 1μm. Note the poly-L-lysine layer. (**B** and **C**) Young MVs prepared without metal coating. Note the heterogeneous size and brightness. Scale bar 5μm. The background noise of some pictures a consequence of the fast scanning speed.

SEM analysis showed the HUVEC-derived MVs to be a heterogeneous population of spherical and elongated structures ranging from 0.2 to 1.8 μm along their longest axis (Fig. 5A). Large MVs were more commonly produced by senescent HUVEC than young HUVEC. Microanalysis revealed the MVs from all sources to have a heterogeneous atomic composition (as demonstrated by the heterogeneous brightness of the MVs; in EDS analysis, elements with a higher atomic number appear brighter because they emit more backscatter signal) (Fig. 5B and C).

**Figure 6:**
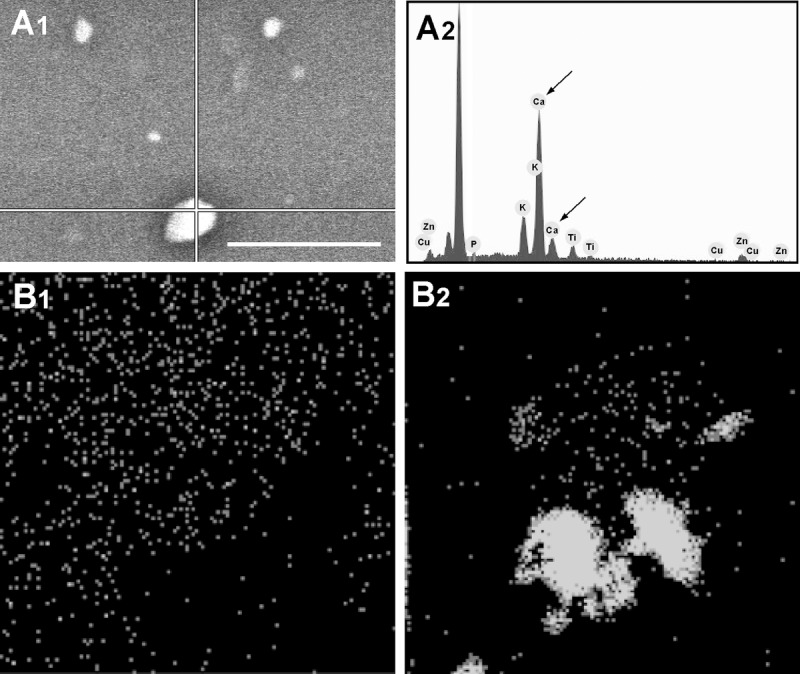
Spot mode microanalysis of an MV (**A1**) with its corresponding plot (**A2**); arrows indicate Ca spikes. Scale bar 5μm. Larger MVs are usually brighter and have a higher Ca content. Ca mapping of MVs from (**B1**) young and (**B2**) senescent HUVEC. Ca is seen as grey on the black background.

To determine the Ca content of the MVs, 60 young and senescent HUVEC-derived MVs were SEM-EDS-analyzed in spot mode. This involves focusing a narrow electron beam at a specific point (spot) on the MV (Fig. 6A). Some 67% of the senescent HUVEC-derived MVs had a high Ca content (i.e., >20% relative content). MVs with low Ca contents (3-10%) represented some 25% of the total number; only 8% had no Ca. In contrast, 84% of the young HUVEC-derived MVs had no Ca; the remaining 16% contained a little Ca. These results agree with the Ca mapping results for both these types of MV (Fig. 6B1 and 2).

**Figure 7:**
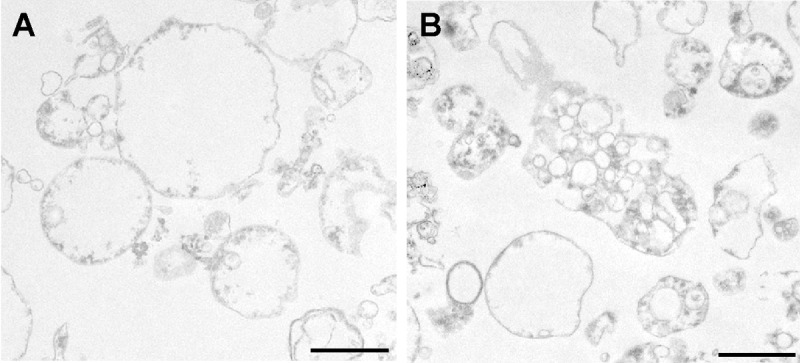
TEM analysis of MVs from (**A**) young and (**B**) senescent HUVEC.

Interestingly, TEM analysis revealed morphological differences between the MVs from the young and senescent HUVEC (Fig. 7). MVs from the former (Fig. 7A) were generally spherical and showed a very simple internal structure. In contrast, few of the MVs from senescent HUVEC were spherical; indeed, a large number of irregular MVs were seen to contain even smaller vesicles of differing electron density (Fig. 7B).

### Senescent endothelial microvesicles contain pro-calcification proteins

**Figure 8:**
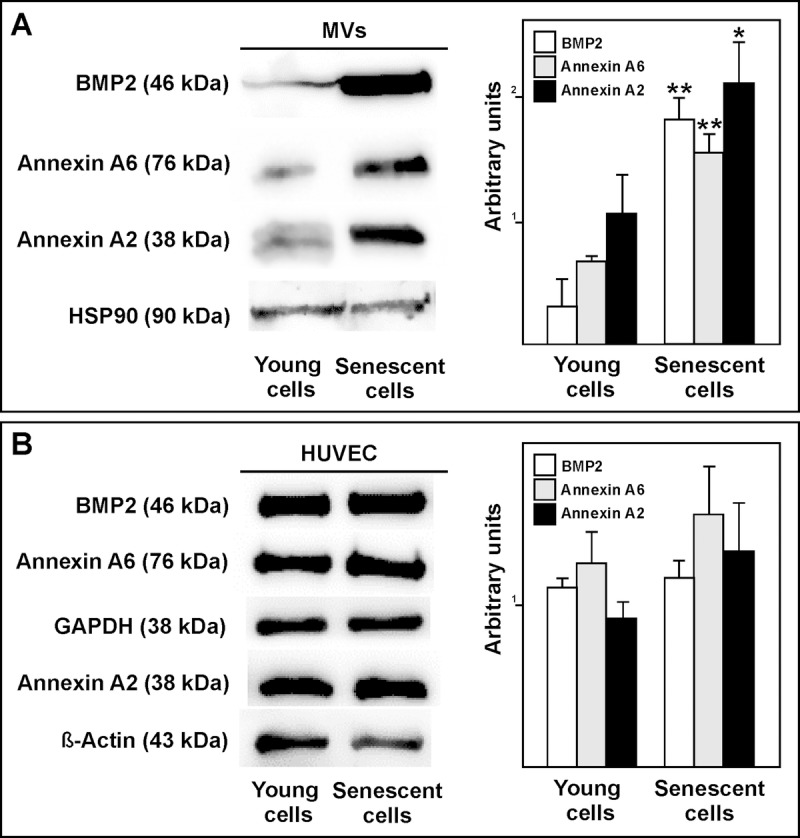
(**A**) Western blot showing that BMP2, annexin A6 and annexin A2 carriage to be higher in MVs from senescent than from young HUVEC. Equal protein loading was confirmed using a HSP90 antibody. Total protein expression was obtained via densitometric analysis and expressed as the ratio protein/HSP90 in arbitrary units. n=3 pools; *p<0.05; **p<0.01. (**B**) Representative BMP2, annexin A6 and annexin A2 western blot of young and senescent HUVEC pools. Equal protein loading was confirmed probing with GAPDH or β-Actin. The graphs present densitometric band analysis normalized to GAPDH or β-Actin in arbitrary units. n=3 pools.

Western blot assays showed the senescent HUVEC-derived MVs to have more annexin A2 and annexin A6 than those from the young cells. Similar results were seen for BMP2 (Fig. 8A). However, there was no change in protein expression of these proteins between young and senescent HUVEC (Fig. 8B). These findings suggest that MVs from senescent endothelial are involved in the appearance of calcification.

## DISCUSSION

It is thought that senescent ECs produce MVs containing compounds that may interfere with the function of vascular cells leading to CVDs [11-13]. In agreement, the present results suggest that the MVs circulating in the plasma of elderly subjects contribute towards the calcification of vascular smooth muscle cells. In addition, those obtained for the *in vitro*-generated HUVEC MVs strongly support the idea that calcification associated with aging is triggered by the MVs produced by senescent ECs. Certainly, the senescent HUVEC MVs contained increased amounts of Ca and bone-associated proteins. They also carried increased amounts of phosphatidylserine (as shown by their marking with annexin A5), and were actively endocytosed by HASMC, which later showed signs of calcification.

Compared to the younger subjects, the plasma of the elderly subjects contained a larger number of MVs in general, and of EC-produced MVs in particular, all cytometrically determined to be about 1 μm in diameter. Other authors have reported similar dimensions for a subset of MVs (microparticles) involved in CVD [11]. Increased numbers of MVs have also been reported in other patients with CVD, supporting the idea that they contribute to its appearance[14]. The present results contrast, however, with those of Forest *et al*.[15] The latter authors reported healthy elderly subjects to produce fewer endothelial MVs. It may be that minor, undetected illnesses, influence the production of MVs.

Only the MVs from the plasma of elderly subjects, and from senescent HUVEC, promoted the calcification of HASMC. It was very difficult to isolate sufficient bona fide EC-derived MVs from the plasma in order to confirm their having a role in vascular calcification. However, more than sufficient such MVs were isolated from senescent HUVEC.

Several studies have reported increased metabolic activity in senescent cells [16-18], which might explain why some of the MVs produced by them were slightly larger. Further, an increased amount of protein in MVs has been reported to be associated with a higher risk of future vascular events and mortality in patients with clinically manifested vascular disease [16-18]. Whether the amount of proteins in these MVs might help identify elderly subjects with increased risk of CVD needs to be evaluated.

The Ca and BMP2 carried by the MVs of senescent HUVEC promote osteoblastic transformation of vascular smooth muscle cells [10, 19]. However, other calcification mechanisms may be involved. Some MVs might act as nodes within the extracellular matrix leading to its direct calcification, while others may enter the smooth muscle cells and then calcify their interior. Certainly, the first of these possible extra mechanisms is used by osteoblasts and chondrocytes to mineralize the extracellular matrix [20]. Intracellular calcification has been documented long time ago [21] and also occurs under physiological conditions in different species [22]. Further, a predominantly intracellular form of calcification has been described to occur in the early stages of calcification in cell cultures [23]. It may therefore be that different MVs use these different mechanisms; the enormous heterogeneity of MVs would appear to support this hypothesis.

It has been reported that calcification-mediating MVs carry extra Ca-binding proteins such as annexin A2 and A6 [6, 7]; this was confirmed in the present work. Annexin A6 is abundant at sites of vascular calcification, a process that is decelerated by siRNA depletion of this protein [7]. Ca-containing MVs have been identified in a range of cells [7, 24, 25], and to be induced by pathological or apoptotic signals. However, the production of Ca-containing MVs by senescent ECs is spontaneous and associated with cell aging.

The present results suggest that, with age, the number of MVs in the plasma increases, promoting vascular calcification. These MVs are likely produced by senescent ECs. Clinical studies are required to determine whether the number of calcifying MVs correlates with vascular calcification in elderly patients, and in those with premature vascular disease. The results also suggest that MVs could be used as markers of vascular calcification; their detection might be used to identify patients at risk of CVD and/or follow the clinical course of their disease. They also suggest that MVs might offer a therapeutic target for the control of vascular calcification and associated CVD.

## METHODS

### Study population

The study subjects were 26 non-smoking volunteers with normal body mass indices. Fifteen were aged 20–28 years, and 11 were aged 75–83 years (Table 1). All were healthy according to analytical criteria and their responses to a medical questionnaire; certainly none had any cardiovascular, autoimmune, metabolic, inflammatory or kidney disease. None took any medication (not even the elderly subjects). The local Scientific Ethics Committee approved the present research protocol. All subjects provided written, informed consent to be included.

### Blood samples

Venous blood samples were drawn from both elderly and younger subjects into EDTA-coated tubes. Platelet-free plasma (PFP) was prepared at room temperature by serial centrifugation (15 min at 1500 x g, followed by 2 min at 13,000 x g). The supernatant was stored in 1.5 mL tubes at −80°C until use.

### Cell cultures

#### Endothelial cells

HUVEC (ATCC Cat No.PCS-100-010) were cultured in endothelial growth medium (EGM) (Lonza) supplemented with 10% heat-inactivated foetal bovine serum (FBS) (Sigma). Cultures were maintained at 37°C in a 5% CO_2_ atmosphere at 95% humidity.

First-passage cryopreserved HUVEC were grown and serially passaged until they reached senescence (the replicative senescence model). Cells passaged <8 times (population doubling [PD]<20.48; with PD calculated as [ln{number of cells harvested} − ln{number of cells seeded})/ln2]) were regarded as young ECs, and those passaged 26-38 times (PD>96.80) were regarded as senescent ECs [26, 27]. In these cells, the proliferation rate is remarkably reduced, and more than 75% are positive for senescence-associated β-galactosidase.

#### Smooth muscle cells

HASMC were obtained from Lonza (cc-2571). Cells were maintained in SmGM-2 medium (Lonza; cc-3181) supplemented with 20% FBS (Sigma) at 37°C in a humidified atmosphere with 5% CO2. HASMC of passage 5– 8 were used in the experiments.

### Isolation, quantification and characterization of microvesicles

Plasma-derived and HUVEC-derived (isolated from the culture medium) MVs were isolated and counted using the quantification method based in the previously published for of the International Society on Thrombosis and Haemostasis [28], with minor modifications. Platelet-free plasma (PFP) was obtained as previous described ("Blood samples" in Methods section). Next, MVs from PFP was isolated by centrifugation (13,000 g for 30 min). Furthermore, MVs from HUVEC were obtained from supernatants were centrifuged at 13,000 g for 30 min as described previously.

Pooled MVs (i.e., from elderly subjects' plasma, from young subjects' plasma, from the medium of young HUVEC cultures, and from that of senescent HUVEC cultures) were characterized in terms of size using a Beckman Coulter Cytomic FC 500 flow cytometer running CXP software. MVs were understood to be those events gated with a size between 0.5-1.5 μm; this gate was established from the side scatter (SSC) *vs*. forward scatter (FSC) dot-plot produced in a standardization experiment using the SPHERO™ Flow Cytometry Nano Fluorescent Size Standard Kit (Spherotech). The latter has size-calibrated fluorescent beads ranging from 0.1-1.9 μm in diameter. Events below 0.2 μm were excluded in order to adequately distinguish true events from the background; events >1.9 μm were excluded to prevent possible confusions with apoptotic bodies. The absolute number of MVs (events) per microliter was determined using Flow Count Calibrator beads (10 μm; Beckman Coulter Inc.) according to the manufacturer's recommendations using CXP software: (MVs counted x standard beads/L)/(standard beads counted). Data were recorded as the mean of three independent measurements of the same sample.

Triple-fluorescent labelling was performed to characterise the protein profile of the plasma MVs and thus determine their cellular origin. This was done by incubating platelet-free plasma (100 μL) with fluorescein isothiocyanate-conjugated (FITC) labelled monoclonal anti-CD31 (BD Pharmingen), peridinin chlorophyll protein complex (PerCP) monoclonal anti-CD42b (Abcam), and phycoerythrin-annexin A5 (BD Pharmingen), together in annexin A5-binding buffer (10 mM HEPES, 7.4 pH, 140 mM NaCl, 2.5 mM CaCl_2_). Isotype negative controls were also prepared. Since CD31 is expressed by both platelets and ECs, but CD42 occurs only in platelets, platelet MVs were defined by events CD31+/CD42+ and endothelial MVs by events CD31+/CD42-. As a control for the annexin A5 labeling, a sample with fluorescein-conjugated annexin A5 using a CaCl2-free solution was established.

MVs from HUVEC culture medium were isolated and characterized with respect to size and in terms of their protein profile using FITC-anti-CD31 and phycoerythrin-annexin A5, as above.

To confirm the exclusion of apoptotic bodies in MVs isolations, the DNA content of the HUVEC-derived MVs was determined by incubating them with acridine orange (Invitrogen) to a final concentration of 20 mM.

### Western blot analyses

Extracts from young and from senescent endothelial cells, and MVs from young and from senescent HUVEC were lysed in CytoBuster Protein Extraction Reagent lysis buffer (Millipore) containing protease and phosphatase inhibitor cocktail (Roche). The total protein content of lysates was quantified using a BCA Protein Assay Kit (Pierce), with BSA as the standard. Briefly, equal amounts of protein (10-50 μg protein/lane) were diluted with reducing sample buffer and separated by SDS/PAGE (10% gel) under reducing conditions. Samples were then transferred onto nitrocellulose membranes (BioRad), blocked in TBS containing 0.1% Tween 20 and 5% dry non-fat milk for 1 h at room temperature, and incubated in the same buffer with different primary antibodies (BMP2, Abcam ab82511, dilution 1:1000, 46 kDa; annexin A6, Abcam ab31026, dilution 1:1,000, 76kDa; annexin A2, Abcam ab41803, dilution 1:1000, 38 kDa). Anti-β-actin (Santa Cruz) (sc-47778, dilution 1:2000, 43 kDa) or GAPDH (Millipore) MAB374, dilution 1:2000, 38 kDa) was used as a loading control for endothelial cells, and anti-HSP90 (Cell Signaling) (CST#4874, dilution 1:1000, 90 kDa) was used as a loading control for MVs. After washing, the membranes were incubated with Novex horseradish peroxidase-conjugated secondary antibodies (1:5000). Bands were visualized with Luminata Crescendo Western HRP substrate (Millipore). The quality of proteins and efficacy of protein transfer was evaluated by Red Ponceau staining. Bands were quantified using Image J software (NIH) and normalized to anti-HSP90 in MVs and anti-GAPDH or anti-β-Actin in HUVEC.

### Calcification Determination

The qualitative staining of HASMC calcification was identified by alizarin red. HASMC (10000 cells/well, 24-well plates) were cultured with 100,000 MVs/mL from the plasma of young or elderly subjects, or with 50,000 MVs/mL from young or senescent HUVEC for 1, 3, 6 and 9 day in 2% FBS-supplemented DMEM. As a positive control, cells were exposure with 2% FBS-supplemented DMEM containing 2.0mM Pi (pro-calcific condition). Culture medium was renewed every 2 days. Detection and quantification of mineral deposition were performed using alizarin red staining. Briefly, samples were fixed (50% ethanol 5 min, 95% ethanol 5 min), stained for 5 min with alizarin red S (40 mM, pH 4.2), rinsed with 50% ethanol, and subsequently scanned.

### Assessment of calcium deposition

After 6 and 9 days of incubation, calcification was quantified. Cells were decalcified with 0.6 N HCl overnight at 4°C. The Ca content of the supernatants was determined by spectrophotometer by a kit containing phenolsulphonephthalein dye (no. DICA-500, QuantiChrom calcium assay kit; BioAssay Systems). Then, the cells were washed three times with PBS (Sigma) and solubilized in 0.1 M NaOH/1% SDS. The protein content was measured using the BCA protein assay kit (Pierce), and the Ca content was normalized for total protein. Ca content of the cells was expressed as μg/mg protein.

### Confocal microscopy

MVs from young and from senescent HUVEC were stained with CellTracker CM-Dil (LifeTechnologies) to visualize interactions between the MVs and HASMC. The latter cells were cultured with the stained MVs for 48 h, washed with PBS, and then stained with phalloidin-FITC (PKH67). They were then fixed with 4% paraformaldehyde solution in PBS, and mounted in gold antifade reagent plus DAPI. Fluorescence was detected using a confocal microscope (Leica Microsystems GmbH).

### Electron microscopy

Clean, dry coverslips (12 mm diameter) were coated with poly-L-lysine (70,000-150,000 Da) using a 0.1% solution of the same (Sigma). A water-repellent circle of approximately 6 mm diameter was then drawn on the coated coverslip using a pap pen. One drop of suspension of MVs from young or senescent HUVEC (6000 MVs/mL) was then placed within this circle and incubated for 1 h in a humidified chamber at room temperature. After a brief wash in PBS, the MVs were fixed in 3% glutaraldehyde for 10 min, rinsed in buffer, dehydrated in an ethanol series and dried following the routine critical point drying procedure for scanning electron microscopy (SEM). For morphological analysis, the prepared MVs were gold-palladium coated (theoretical thickness 5 nm) in a Polaron E5400 metal evaporator (BioRad), and examined using a Zeiss DSM 950 SEM.

For Ca microanalysis, non-metal-coated MVs were prepared using a Hitachi TM-1000 SEM with an EDS system. For Ca mapping, MVs samples were fixed in 70% ethanol and deposited on a formvar-coated copper grid and analysed using a Zeiss LEO 906E transmission electron microscope (TEM) equipped with an elemental analysis environmental module.

For ultrastructual analysis, samples were fixed in 2.5% (v/v) glutaraldehyde and 0.05% (w/v) ruthenium red in 0.05 M sodium cacodylate buffer for 4 h at 4°C. They were then post-fixed with 1% (w/v) OsO_4_ in 0.1 M sodium cacodylate buffer for 1 h at room temperature, dehydrated in ethanol, and infiltrated with resin. Ultrathin sections were cut using a Reichert Jung E ultramicrotome. Finally, the sections were stained for 10 min with 1% (w/v) aqueous uranyl acetate solution and lead citrate, and viewed using the same TEM as above.

### Statistical analysis

Data are expressed as means±SEM and analysed by ANOVA followed by the Duncan test. Comparisons between pairs of means were performed using the Student t test or Mann–Whitney U test as required. Significance was set at p<0.05.
